# Genetic Variation Interacts with Selenium Exposure Regarding Breast Cancer Risk: Assessing Dietary Intake, Serum Levels and Genetically Elevated Selenium Levels

**DOI:** 10.3390/nu14040826

**Published:** 2022-02-16

**Authors:** Malte Sandsveden, Ylva Bengtsson, Olle Melander, Ann H. Rosendahl, Jonas Manjer

**Affiliations:** 1 Department of Clinical Sciences Malmö, Lund University, 20213 Malmö, Sweden; ylva.bengtsson@med.lu.se (Y.B.); olle.melander@med.lu.se (O.M.); jonas.manjer@med.lu.se (J.M.); 2 Department of Surgery, Skåne University Hospital, 20501 Malmö, Sweden; 3 Department of Internal Medicine, Skåne University Hospital, 20501 Malmö, Sweden; 4 Department of Clinical Sciences Lund, Oncology, Lund University and Skåne University Hospital, 22184 Lund, Sweden; ann.rosendahl@med.lu.se

**Keywords:** selenium, breast cancer, single-nucleotide polymorphisms, cohort

## Abstract

Selenium has been suggested to be protective regarding breast cancer risk but no overall effect has been established. Genetics may modify the effect. This study compares the effect of selenium exposure on breast cancer risk between women with different alleles in single-nucleotide polymorphisms (SNPs). The Malmö Cancer and Diet Study, a cohort including 17,035 women and >25 years of follow-up on breast cancer diagnosis, was used. Five promising SNPs regarding interaction with selenium exposure were selected from the literature: rs1050450, rs4880, rs3877899, rs7579, and rs71304. Selenium exposure was assessed in three ways: genetically elevated (*n* = 16,429), dietary intake (*n* = 15,891) and serum levels (*n* = 2037) at baseline. Cox regression and logistic regression analyses evaluated breast cancer risk from selenium exposure, stratified for the SNPs and adjusted for risk factors. A total of 1946 women were diagnosed with breast cancer. Women with T/T alleles in rs1050450 had lower breast cancer risk compared with C/C, HR 0.81 (0.68–0.96). Interaction by rs1050450 limited a protective effect of higher selenium intake to T/T carriers, HR 0.68 (0.43–1.08) for intermediate intake and HR 0.63 (0.40–1.00) for high intake. No interactions or risk differences were seen for other SNPs or for serum selenium or genetically elevated selenium. The results indicate that genetic variation in rs1050450 might affect breast cancer risk and that selenium exposure could be a possible modifiable risk factor for breast cancer among women with that variation.

## 1. Introduction

Due to its significant role in antioxidation, the essential mineral selenium has been of interest to study in cancer development, including breast cancer [1]. Current evidence does not support a general association between selenium levels and breast cancer risk, although individual studies have found an inverse relationship [2,3,4]. However, evidence suggests that the effect of genetic variation through single-nucleotide polymorphisms (SNPs) in selenium-associated genes needs to be considered [5,6]. Selenium mainly exerts its biological effect through selenoproteins, a group of 25 human proteins exclusively incorporating the selenium-containing amino acid selenocysteine [7]. Many selenoproteins are antioxidative enzymes, such as glutathione peroxidase-1 (GPX-1) that reduces intracellular hydrogen peroxide, and gluthathione peroxidase-4 (GPX-4), a membrane-bound selenoprotein important for lipid peroxidation [8]. The activity of selenoproteins is affected by selenium status [9]. Indeed, the antioxidant function of selenoproteins has been suggested as the probable mechanism involved in the association of selenium and cancer development [8]. The SNP rs1050450 in GPX-1 as well as the SNP rs4880 in superoxide dismutase 2 (SOD-2), a related antioxidative enzyme that is not a selenoprotein, have been reported to modify the association of selenium exposure on cancer development, indicating that some individuals might be at higher risk than others [10,11]. A direct association between breast cancer risk and these two SNPs has also been reported [12,13]. Other SNPs with similar potential have been described, e.g., rs3877899 and rs7579 in selenoprotein P (SEPP1) and rs713041 in GPX-4 [12,14,15].

Recently, several large studies on breast cancer risk have been performed using different selenium exposure measurements. No association between genetically elevated levels of circulating selenium and breast cancer risk was found in a recent Mendelian randomization (MR) study [16]. Null results were also seen when comparing quartiles of dietary selenium intake [17]. In line with these results, our group reported equal breast cancer risk across serum selenium quartiles [18]. However, these studies did not consider modification by genetics. Furthermore, an effect of selenium exposure might only be seen in areas with low selenium intake since selenoprotein activity is increased by selenium intake, but only up until 49–75 μg/day [19]. Intake recommendations in nutritional guidelines are often calculated to reach saturated GPX or SEPP levels, and are in the range of 26–70 μg/day [20,21,22]. An adult woman in Sweden is recommended 50 μg/day [20]. In the study regarding dietary intake mentioned above, the average selenium intake was 103 μg/day and the authors indeed state that their results are only applicable to a population with sufficient selenium intake [17]. However, recent results published by our group from a low-selenium setting (mean selenium intake 43 μg/day) similarly found no evidence of an overall association, but indicated a u-shaped association, with the lowest risk of breast cancer among women with intermediate intake of selenium, and interactions with BMI and smoking, attenuating the u-shape among smokers and overweight women [23]. This suggests that the underlying selenium status is important, but other factors might modify the association.

The aim of this study was to determine whether the effect of selenium exposure on breast cancer risk is modified by relevant SNPs, and whether any independent effect of these SNPs on breast cancer risk exists, in a low-selenium setting. Selenium exposure was assessed by applying a triangulation approach encompassing a genetic score for higher selenium levels, dietary selenium intake and serum selenium levels. Five SNPs (rs1050450, rs4880, rs3877899, rs7579 and rs713041) were evaluated regarding effect modification.

## 2. Materials and Methods

### 2.1. The Malmö Diet and Cancer Study (MDCS)

This study was designed within the MDCS, a population cohort based in the Swedish city of Malmö. As previously described, citizens of Malmö aged 44 to 74 were invited to the cohort and participants were included from 1991 to 1996 [24]. In total, 43% of eligible participants were included, of which 17,035 were women. At baseline, blood samples were drawn and dietary data were collected. Participants provided height and weight measurements and answered a questionnaire regarding, e.g., education, occupation, diseases, lifestyle and reproduction [24]. Information regarding breast cancer risk factors collected at baseline is presented in Table 1.

### 2.2. Inclusion and Exclusion

**Figure 1:**
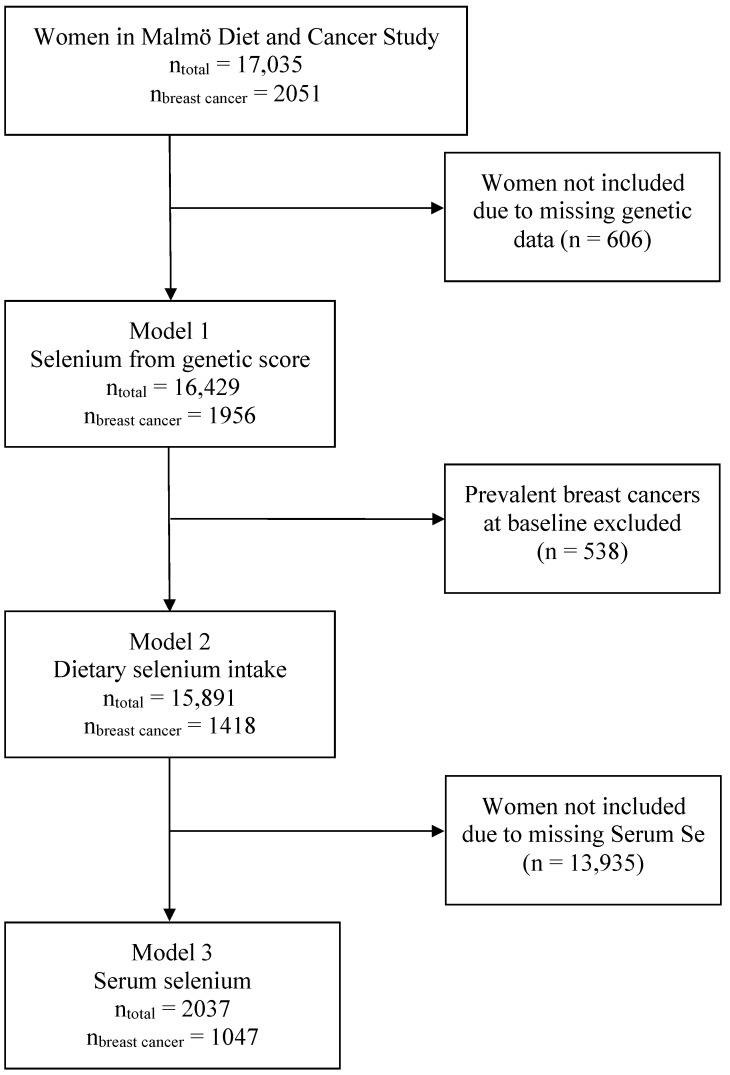
Inclusion and exclusion flowchart.

Inclusion and exclusion were different for each of the three exposure assessments and are visualized in Figure 1. Women in MDCS with valid genotyping were included in the analyses based on genetic score (*n* = 16,429). Women with prevalent breast cancer at baseline (*n* = 538) were then excluded from the analyses based on dietary selenium intake (*n* = 15,891). Only women with valid data regarding serum selenium were then included for analyses based on serum levels (*n* = 2037). Serum selenium values were available from a case–control study within the MDCS from 2017, including incident breast cancer cases to 31 December 2013 (*n* = 1186) and an equal number of controls [18]. The control selection is described in the original article and included both an incidence density matching (based on age, time at inclusion and menopausal status) from an earlier work by Almquist et al. (2010) and a randomization of the remaining controls by Sandsveden and Manjer (2017) [18,25].

### 2.3. Genotyping

The genotyping in MDCS has been described previously [26]. Stored serum samples from the MDCS were analyzed with an Illumina GSA v1 genotyping array. Quality control included removal of individuals with <90% call rate, sample duplicates and individuals that differed between genetically inferred and reported sex. Genetic variants not in the Hardy–Weinberg equilibrium (*p* < 1 × 10^−15^) were removed. Missing SNPs were imputed using a reference panel from the Haplotype Reference Consortium [27]. Genetic data were available for 16,429 out of the 17,035 women in the MDCS who completed baseline examinations. The number of alternative alleles (0–2) was represented by a continuous value in the range 0–2, representing the number measured through genotyping or the imputed probability. The data from imputation were centered around 0, 1 and 2 as shown in Supplementary Figure S1 and the data were categorized as 0, 1 or 2 alleles (<0.5, 0.5–1.5, ≥1.5).

### 2.4. Selection of Candidate SNPs for Interaction

Five SNPs with a reasonable mechanistic pathway and evidence of effect on selenoproteins and/or breast cancer development were identified in previous research (rs1050450, rs4880, rs3877899, rs7579 and rs713041) [10,11,12,13,14,15].

### 2.5. Allele Score for Genetically Elevated Selenium

The allele score design was adopted from the MR study on predicted levels of circulating micronutrients and breast cancer risk by Papadimitriou et al. (2021), and each SNP was weighted for its effect on selenium levels (see their Supplementary Table S1) [16]. The SNPs were selected from two genome-wide association studies of serum and toenail selenium, by using the commonly accepted threshold of *p* = 5 × 10^−8^ and excluding SNPs in linkage disequilibrium (LD r2 ≤ 0.1), as well as one SNP, rs6859667, with a minor allele frequency less than 5% [16,28,29]. The following SNPs were used to construct the weighted allele score in the present study: rs921943, rs567754, rs3797535, rs11951068, rs705415, rs6586282, and rs1789953. The data in the MDCS dataset were harmonized so that all alleles in the allele score were instruments for increased serum selenium. The allele score was then split into tertiles by rank, representing increasing effects on serum selenium levels.

### 2.6. Dietary Selenium Intake

The dietary data used in the present study are total intake of energy and selenium, including intake from diet and supplements. The method used for data collection has been described previously [30]. In short, the method consisted of three parts that each participant of MDCS completed at inclusion: (1) a semi-quantitative questionnaire regarding food and supplement intake, dietary habits and portion sizes based on the preceding year; (2) a diet diary including documentation of all food and beverage intake during a seven-day period; (3) an interview lasting 45–60 min to further specify portion sizes and meal preparations. Average daily consumption of different foods was then calculated and converted into intake of specific nutrients by using the Swedish Food Database PC KOST2 -93 from the Swedish National Food Administration.

The association of nutrients to risk of disease is often biased by the correlation to total energy intake; therefore, adjustment for energy intake is needed [31]. In the present study, the nutrient residuals method was used. Based on a linear regression analysis of total selenium intake and total energy intake each individual was assigned their residual value (distance from the regression line). The residual variable was then split into tertiles by rank. The ranking was performed separately for the season of dietary data collection (January–March, April–June, July–September, and October–December) to adjust for seasonal intake differences.

### 2.7. Serum Selenium

Serum selenium was analyzed in a previous study, with methodology described in more detail [18]. In short, 0.15 mL serum from stored baseline samples was thawed and selenium analyzed using inductively coupled plasma sector field mass spectroscopy. The coefficient of variation was 0.03 for inter-batch variation. Women with incomplete analyses in that study (*n* = 262) were not included in the serum selenium analyses in the present study. The serum selenium variable was split into tertiles by rank, and this was performed separately for each season as described above.

### 2.8. Endpoint

The Swedish personal number was used to cross-link MDCS to registry data up until 31 December 2019. Breast cancer diagnosis including the date of diagnosis was identified in the Swedish Cancer Registry, date of death was identified in the Swedish Cause of Death Registry, and emigration was identified in The Swedish Population Registry.

### 2.9. Missing Values

Missing values were explored among the 15,891 women included in the dietary intake analyses and considered to be missing at random. A total of 96.0% had complete data with no missing values. The variable with the most missing values was ‘Age at first childbirth’ including 269 women (1.7%) with missing information. The missing values were imputed using chained equations. Predictive mean matching was chosen as the model type for scale variables. In total, 25 new datasets, each with 15,891 individuals, were created from 10 iterations each. The multiple imputation model included all factors in the dietary intake analyses—endpoint, time at risk (logarithmic), exposure (residual of selenium intake and season), risk factors (Table 1) and rs1050450, rs4880, rs3877899, rs7579, and rs713041. The original data and the pooled imputed data are presented in Supplementary Table S1. This multiple imputation model was used both for analyses based on dietary selenium intake and serum selenium. For sensitivity analysis, a separate imputation model was constructed including only the 2037 women with valid serum selenium, and replacing the residual value of selenium intake with serum selenium. A sensitivity analysis with only complete cases was also performed to evaluate the robustness of the imputation model.

### 2.10. Statistical Analysis

Descriptive statistics for tertiles of allele score in relation to established risk factors for breast cancer were investigated in a cross table. Descriptive statistics for dietary selenium intake and serum selenium have been published previously for this cohort [18,23].

Kaplan–Meier curves were used to evaluate the assumption of proportional hazards for dietary intake and genetically elevated selenium. Cox regression analyses were used to evaluate the risk of breast cancer and render HRs and 95% confidence intervals. In analyses considering genetically elevated selenium, allele score tertiles were compared, using ‘low’ as the reference. The underlying time variable was age at censoring. The indicator of event was a first-time breast cancer diagnosis, including diagnoses both before and after baseline of MDCS. Other reasons for censoring were death, emigration or end of follow-up. The analyses were also stratified for the included SNPs (rs1050450, rs4880, rs3877899, rs7579, and rs713041); and in a separate analysis, an interaction term of the allele score and the SNP variable was included along with the two variables separately. Separate Cox regression analyses were performed for rs1050450, rs4880, rs3877899, rs7579 and rs713041, comparing the number of effect alleles (0, 1, and 2) and the risk of breast cancer.

In analyses based on dietary selenium intake, only a first-time breast cancer diagnosis after baseline was used as the indicator of event and, consequently, the time at risk started at the cohort baseline and the time in cohort was used as the underlying time variable. Analyses compared tertiles of dietary selenium intake and were first adjusted for age at baseline, and then for additional established risk factors for breast cancer; age at menarche, age at menopause, BMI, education, socio-economic status, marital/cohabiting status, number of children, age at first childbirth, ever use of contraceptive pills, oophorectomy, alcohol consumption and use of hormone replacement therapy. Analyses were stratified for SNPs and interaction was tested as described above. Sensitivity analyses including only complete cases were also performed.

The analyses based on serum selenium were treated as a case–control design. Hence, tertiles of serum selenium levels were compared with logistic regression, rendering odds ratios (ORs) regarding risk of incident breast cancer from baseline until end of follow-up. Analyses were adjusted for the same factors as the dietary intake analyses. Analyses were stratified and interaction was tested as described above. Sensitivity analyses were also performed, using the multiple imputation based on women with valid serum selenium as described above.

All analyses were performed in IBM SPSS 27.0.1.0.

## 3. Results

Descriptive statistics for the included and excluded women are presented in Table 1. Women with prevalent breast cancer at baseline were older than the other groups at baseline, less likely to have children and older at first childbirth. Women who had a first-time breast cancer diagnosis during the cohort follow-up were younger than women without breast cancer and were also less likely to have children but had a higher alcohol intake and were more likely to be non-manual workers and more likely to have used oral contraceptives, but were less likely to use hormone replacement therapy at baseline.

Descriptive statistics for low, intermediate and high allele scores are presented in Supplementary Table S2. Risk factors for breast cancer were similar across all tertiles.

In Supplementary Figure S2, Kaplan–Meier curves comparing tertiles of selenium exposure and breast cancer risk are presented. There was no evidence of differences in breast cancer risk between tertiles of allele score. Women with intermediate selenium intake had a lower breast cancer risk compared to women with high or low selenium intake, and the hazards were considered proportional.

### 3.1. Breast Cancer Risk from Genetically Elevated Selenium and Individual SNPs

**Figure 2:**
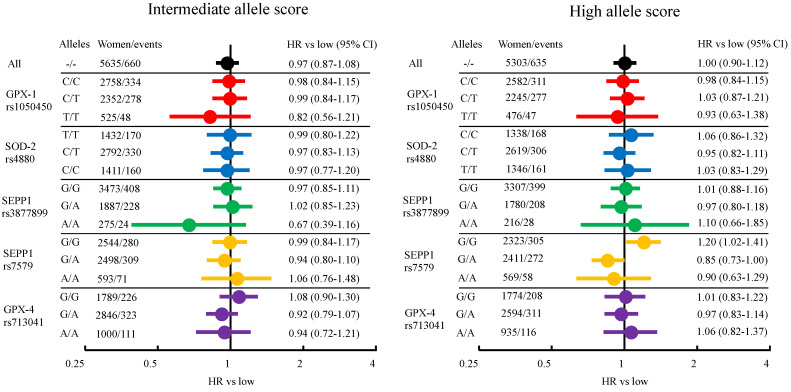
Forest plot of unadjusted hazard ratios (HR) for allele score tertiles and breast cancer risk. Each color represent results stratified for different SNPs.

When comparing tertiles of allele score, 16,429 women were at risk for breast cancer from birth to the end of follow-up, resulting in a mean follow-up time of 80.0 years, a total of 1,280,698 person years (py) and 1956 individuals with a breast cancer diagnosis. In Figure 2, HRs comparing the allele score tertiles are presented in a forest plot. Full data are presented in Supplementary Table S3. In Table 2, the HRs for breast cancer risk for each SNP are presented. There were no overall differences in breast cancer risk among the tertiles of allele score, HR low: 1.00 (reference), HR intermediate: 0.97 (0.87–1.08) and HR high 1.00 (0.90–1.12). In analyses stratified by SNPs, an interaction was seen between the allele score and rs7579. Women with G/G alleles and a high allele score had a HR of 1.20 (1.02–1.41) compared to low, while G/A had 0.85 (0.73–1.00) and A/A 0.90 (0.63–1.29). Both the highest and the lowest absolute risks of breast cancer when comparing tertiles of allele score were found in the stratum including women with A/A alleles in rs3877899, 171/100,000 py for women with a high allele score, and 111/100,000 py for women with an intermediate allele score. However, only 28 and 24 cases were reported in these groups, respectively. When comparing individual SNPs and breast cancer risk, the lowest absolute risk (127/100,000 py) was found among women with T/T alleles in rs1050450, HR 0.81 (0.68–0.96), compared to women with C/C alleles (156/100,000 py). No differences in breast cancer risk were seen for the other SNPs.

### 3.2. Breast Cancer Risk from Dietary Selenium Intake

**Figure 3:**
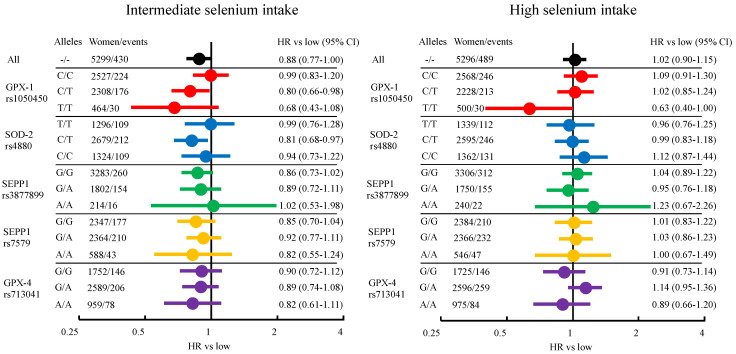
Forest plot of age adjusted hazard ratios (HR) for selenium intake tertiles and breast cancer risk. Each color represent results stratified for different SNPs.

When comparing tertiles of dietary intake, 15,891 women were at risk of breast cancer from inclusion in MDCS to the end of follow-up or censoring. With a mean follow-up time of 21.5 years, and a total of 342,129 py, 1418 women were diagnosed with breast cancer. In Figure 3, age-adjusted HRs are presented in a forest plot, while HRs adjusted for additional known breast cancer risk factors are presented in Supplementary Table S4. An overall lower risk of breast cancer was seen among women with intermediate intake of selenium, HR 0.88 (0.77–1.00) for age-adjusted analysis and HR 0.86 (0.76–0.98) when adjusted for additional risk factors, compared to those with low intake. Risks were similar for low and high intake. In stratified analyses, an interaction was seen between selenium intake and rs1050450. In the stratum of women with T/T, there was a lower risk of breast cancer among women with intermediate selenium intake, age-adjusted HR: 0.68 (0.43–1.08), and high intake of selenium, age-adjusted HR: 0.63 (0.40–1.00), compared to low. However, that stratum had too few events for adjusting for the additional breast cancer risk factors (47 in low, 30 in intermediate, 30 in high). There were small differences in point estimates between age-adjusted and additionally adjusted analyses. The largest effect was seen among women with rs1050450 C/T and high selenium intake (age-adjusted HR 1.02, additionally adjusted HR 0.95). The absolute risk of breast cancer when comparing dietary selenium intake was highest for women with rs713041 A/A and low selenium intake, and for women with rs713041 G/A and high selenium intake (466/100,000 py), while it was lowest for women with rs1050450 T/T and high selenium intake (278/100,000 py).

### 3.3. Breast Cancer Risk from Serum Selenium

**Figure 4:**
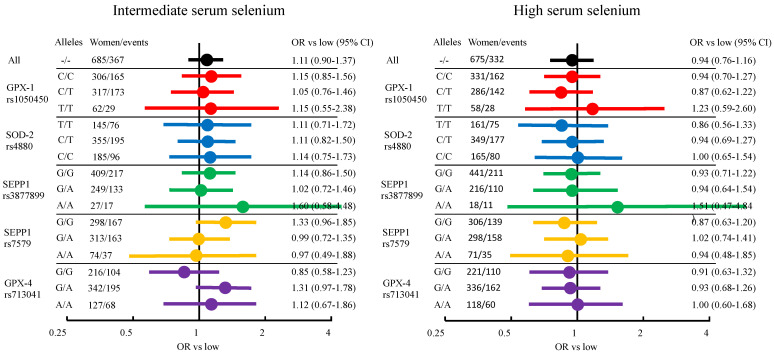
Forest plot of age adjusted odds ratios (ORs) for serum selenium tertiles and breast cancer risk. Each color represent results stratified for different SNPs.

In total, 2037 women with data on serum selenium levels were followed for a mean time of 17.0 years from baseline. During follow-up, 1047 out of those women were diagnosed with breast cancer. No overall differences between tertiles or any interactions with individual SNPs were seen. In Figure 4, age-adjusted ORs are presented in a forest plot, while ORs adjusted for additional breast cancer risk factors are presented in Supplementary Table S5.

In the complete case analyses, results were similar to adjusted analyses (results not shown). In the sensitivity analyses for serum selenium, using the alternative multiple imputation model, the adjusted ORs were similar (results not shown).

## 4. Discussion

The two main findings in this study were that rs1050450 in GPX-1 is associated with decreased risk of breast cancer for T/T carriers, and modifies the effect of dietary selenium intake on breast cancer risk. A protective effect of intermediate or high selenium intake was seen among women carrying C/T or T/T alleles in a stepwise fashion, compared to women with the C/C allele. The overall effect of selenium intake and serum selenium has previously been reported for this cohort, and we found no further interactions with the other four SNPs investigated [18,23]. We found no effect on breast cancer risk when using genetically elevated selenium levels as exposure measurement.

Loss of heterozygosity in the GPX-1 gene is a common event in breast cancer, as well as in other cancers, and suggests an important role for GPX-1 in cell homeostasis [10,32]. GPX-1 is one of the selenoproteins whose levels decrease the most in selenium deficiency, making it susceptible to selenium status [9]. The SNP rs1050450 gives rise to two different functional variants of GPX-1; the more common C allele leads to proline at codon 198 while a T allele leads to leucine [33]. The effect of rs1050450 is not fully understood on a cellular level. One study suggests that leucine increases the cytoplasmic proportion of GPX-1, consequently increasing reactive oxygen species in the mitochondria [34]. SNPs in two other selenoproteins, SEPP1 (rs387789 and rs7579) and GPX-4 (rs713041), were also studied. SEPP1 functions mainly as a selenium transporter, while GPX-4 is important in lipid peroxidation [8,35]. SOD-2 is not a selenoprotein, but its antioxidative activity is essential for the levels of reactive oxygen species in human cells and is intimately connected with GPX-1 [36,37].

Our results are in line with findings from a study including 136 Polish women with breast cancer where women carrying T/T or C/T in rs1050450 had lower risk compared to C/C [14]. In contrast, results from a Danish cohort indicated higher risk for women with T alleles in rs1050450 [38]. However, updated results from the same cohort found an increased risk only for non-ductal breast cancer (*n* = 190), odds ratio (OR): 1.88 (1.08–3.28), while the results were more in line with the present study for ductal breast cancer (*n* = 659) with an OR: 0.73 (0.47–1.14) and for overall breast cancer (*n* = 975) with an OR: 0.83 (0.54–1.28) [12]. Other studies have found null results, and a meta-analysis by Hu et al. (2010) found no overall association for women of Caucasian heritage, although it did not include the studies mentioned above because they were published after the meta-analysis [39]. A lower risk of breast cancer for women with the T allele in rs1050450 was found in a GWAS study including 280,000 women [40]. However, in a study from the Breast and Prostate Cancer Cohort Consortium, including 10,000 women with breast cancer and 7500 men with prostate cancer, a lower risk of prostate cancer but not breast cancer was seen for T/T carriers [41]. In a meta-analysis regarding rs1050450 and overall cancer risk from 2017, 21,000 cancer patients were included, of which 7800 were women with breast cancer. In that study, no difference in breast cancer risk was found when comparing different alleles; however, an increased risk of any cancer and bladder cancer for T/T carriers was seen [42]. Thus, our results regarding an increased risk of breast cancer for women with the T allele in rs1050450 are in line with the largest study so far, but results from other studies and regarding other cancer types vary.

Regarding the interaction of rs1050450, one study found that women with T/T had the lowest levels of lipid peroxidation and that the GPX-1 activity was associated with lipid peroxidation, but that selenium levels did not affect the association [14]. Those results were inconsistent with previous findings that supported a stronger correlation between serum selenium levels and GPX-1 activity among individuals with C/C compared to T/T among 405 patients hospitalized for non-cancer diagnoses [43]. Furthermore, T alleles lead to lower GPX-1 activity in response to selenium in vitro [44]. Although, intuitively, higher GPX-1 activity could suggest better protection against oxidative stress and thus cancer, the relationship is complex. As discussed by Schumacker (2006), increased protection against oxidative stress is not only beneficial, but paradoxically also important for malignant cells to avoid apoptosis in a pro-oxidative environment [45]. In the present study, there was also evidence of an interaction between rs7579 and the allele score. It was an isolated finding, with no evidence of a dose–response or stepwise association, and was thus possibly a chance finding due to multiple testing in a limited population.

### Strengths and Limitations

Challenges of observational studies include validly measuring exposure and outcome. Outcome data were collected from the Swedish Cancer Registry with high validity and completeness [46]. Limitations in exposure measurements include that recollection of dietary intake may be imprecise and that selenium is difficult to isolate from energy intake and other nutrients since they it is consumed as part of a dietary pattern [31]. The dietary assessment in MDCS showed good ranking validity compared to weighted food records and the residual method adjusts for energy intake [47]. Selenium content can differ between two comparable food items depending on origin, leading to inaccuracy of measurement, and intake does not reflect effects from metabolic processes [48]. Using serum selenium for exposure measurement somewhat avoids these limitations. Serum selenium adequately predicts GPX levels but has a low correlation to other selenium specifications, and is also correlated to other factors such as smoking, BMI and age [49]. Thus, risk of exposure misclassification is hard to avoid completely in designs including dietary intake and serum selenium. The allele score theoretically further avoids risk of bias due to randomization at birth, and our individual-level data indicated good random sampling with even distribution of breast cancer risk factors over allele score tertiles. However, the allele score only accounts for a small proportion of the total variation in selenium, and statistical power can be an issue. A previous study reported that their weighted allele score accounted for 2.5–5% of serum selenium levels [50]. The authors of the MR study that was the source of our weighted allele score estimated the proportion explained by the allele score to be 3% and they had 98% statistical power for finding an OR of 1.10 per 1 SD change in selenium exposure based on 122,977 cases and 105,974 controls compared to our 1956 cases and 14,473 controls [16]. A possible reason for not detecting a correlation with MR design could also be a u-shaped association, as our dietary data suggest. However, performing sub-group analyses would decrease the statistical strength further and no conclusions could be drawn from such analyses in our data. A strength of the present study is that it applied a triangulation approach with three different measurements of selenium exposure [51].

For rare alleles in some SNPs, the number of events was low in the present study. That resulted in zero events in some categories when adjusting the Cox regression for all breast cancer risk factors, and those analyses were discarded. Cox regression analyses should be interpreted with caution if <10 events per category, although most analyses are stable for up to five events per category [52]. Across all categories, results were similar in age-adjusted analyses and those adjusted for additional risk factors.

The reason for including prevalent breast cancers as endpoint in the model evaluating genetically elevated selenium but not in the models evaluating dietary intake and serum levels was to avoid the risk of reverse causation, since there is a risk that a prevalent breast cancer disease could have affected the dietary intake and the serum selenium levels, as well as some of the risk factors evaluated at baseline. However, prevalent disease at baseline would not affect the studied genetics, and thus there is no risk of reverse causation.

## 5. Conclusions

Our findings support a lower risk of breast cancer for women with the SNP rs1050450 T/T in GPX-1, and a protective effect of dietary intake of selenium among these women. However, due to few events and no evidence of a similar effect with regard to serum selenium or genetic score, the evidence is weak and our findings should be seen as indications and need to be replicated in other cohorts. However, our findings are in line with ideas presented in recent reviews, and further mechanistic and epidemiological studies are warranted since selenium exposure could be a modifiable risk factor for some women.

## Figures and Tables

**Table 1:** Established and potential risk factors for breast cancer among included and excluded women.

		No Breast Cancer (*n* = 14,473)	Incident Breast Cancer (*n* = 1418)	Breast Cancer at Baseline (*n* = 538)	No Genetic Data (*n* = 606)
		Valid Column %	Valid Column %	Valid Column %	Valid Column %
Age at baseline * (SD)		57.4 (7.9)	56.0 (7.3)	60.7 (7.4)	57.1 (7.3)
Age at menarche * (SD)		13.6 (1.5)	13.5 (1.4)	13.7 (1.4)	13.6 (1.5)
Parity	0	12.7	14.0	16.7	14.8
	1	21.7	19.5	28.4	22.5
	2	41.5	46.5	35.8	41.9
	3	17.2	15.1	13.4	13.0
	4 or more	6.8	4.8	5.7	7.9
	Missing	1.6	2.0	1.9	3.8
Ever use of oral	Never	51.1	45.3	56.8	54.1
contraceptives	Ever	48.9	54.7	43.2	45.9
Married or cohabiting	No	33.1	31.5	36.2	36.5
	Yes	66.9	68.5	63.8	63.5
Bilateral	No	98.6	98.6	98.0	97.7
oophorectomy	Yes	1.4	1.4	2.0	2.3
BMI	BMI < 25	53.7	51.0	50.4	50.4
	BMI 25–30	32.7	35.2	35.5	35.3
	BMI ≥ 30	13.5	13.8	14.1	14.3
Education	O-level college	70.0	67.3	72.6	69.2
	A-level college	7.1	6.5	5.4	6.8
	University	23.0	26.2	22.0	24.0
Socio-economic index	Manual	38.6	33.4	37.2	38.7
	Non-manual	53.7	59.7	57.0	53.8
	Employer	7.7	6.9	5.8	7.5
	Missing	1.0	1.1	1.5	1.5
HRT use at baseline	No	82.4	73.7	95.0	81.3
	Yes	17.6	26.3	5.0	18.7
Alcohol intake	No alcohol	7.8	5.6	9.1	5.8
	<15 g/day	64.2	62.8	61.5	65.8
	15–30 g/day	14.0	15.5	13.0	12.7
	>30 g/day	2.2	4.2	3.4	2.1
	Infrequent use	11.8	12.0	13.0	13.5
Age at menopause **	Hysterectomy	0.5	0.6	0.9	0.5
	Pre-/peri	33.3	41.5	17.6	31.7
	≤44	10.1	8.5	8.2	12.9
	45–54	50.2	43.7	62.1	49.6
	≥55	5.9	5.8	11.2	5.3
	Missing	0.7	0.8	0.6	1.2
Age at first childbirth	≤20	17.1	15.9	13.3	18.4
	21–25	36.0	35.2	31.3	34.0
	26–30	24.8	24.5	27.3	22.5
	≥31	9.3	10.4	11.6	10.5
	Nullipara	12.8	14.0	16.7	14.8
	Missing	1.7	2.0	1.9	3.8

Values are valid column % except for missing values that are total column %. Missing is not presented if <1% missing in all columns. * Age is presented as mean years and standard deviation (SD). ** Hysterectomized or pre-/perimenopausal women at baseline in separate categories. HRT: hormone replacement therapy.

**Table 2:** Unadjusted hazard ratios (HR) with 95% confidence intervals (CI) for risk of breast cancer comparing breast cancer events for women with 1 or 2 effect alleles to women homozygote for reference allele of different SNPs.

		Women with Genetic Data (*n* = 16,429)		
SNP	Alleles	Events/Women (%)	Events/ 100,000 py	HR (95% CI)
GPX-1	C/C	965/7951 (12.1%)	156	1
rs1050450	C/T	843/6988 (12.1%)	155	0.99 (0.90–1.08)
	T/T	148/1490 (9.9%)	127	0.81 (0.68–0.96)
SOD-2	T/T	498/4113 (12.1%)	156	1
rs4880	T/C	980/8216 (11.9%)	153	0.98 (0.88–1.09)
	C/C	478/4100 (11.7%)	149	0.96 (0.84–1.08)
SEPP1	G/G	1220/10208 (12.0%)	153	1
rs3877899	G/A	655/5494 (11.9%)	153	1.00 (0.91–1.10)
	A/A	81/727 (11.1%)	144	0.95 (0.76–1.19)
SEPP1	G/G	864/7370 (11.7%)	150	1
rs7579	G/A	898/7328 (12.3%)	157	1.04 (0.95–1.14)
	A/A	194/1731 (11.2%)	144	0.96 (0.82–1.12)
GPX-4	G/G	651/5405 (12.0%)	154	1
rs713041	G/A	959/8089 (11.9%)	152	0.99 (0.90–1.10)
	A/A	346/2935 (11.8%)	151	0.98 (0.68–1.12)

py = person years.

## Data Availability

Data will be made available from the corresponding author on a reasonable request.

## Supplementary Materials

The following supporting information can be downloaded at: https://www.mdpi.com/article/10.3390/nu14040826/s1, Supplementary Tables S1–S5 and Supplementary Figures S1–S2.
