# Supplementary material for: Genetic Variation Interacts with Selenium Exposure Regarding Breast Cancer Risk: Assessing Dietary Intake, Serum Levels and Genetically Elevated Selenium Levels
Source: Nutrients. 2022 Feb 16;14(4):826. doi: 10.3390/nu14040826 (PMC8875528; doi:10.3390/nu14040826)

Supplementary Table S1. Pooled imputed values and original values.

		Imputed (N=397,275)	Original (N=15,891)
		Valid column %	Valid column %
Age at baseline (mean years)		57.3	57.3
Age at menarche (mean years)		13.6	13.6
Ever use of oral contraceptives	never	50.6	50.6
	ever	49.4	49.4
	missing	0.0	0.0
BMI	BMI <25	53.5	53.5
	BMI 25-30	33.0	33.0
	BMI ≥30	13.6	13.6
	missing	0.0	0.1
Alcohol intake	no alcohol	7.7	7.6
	<15 g/day	64.1	64.1
	15-30 g/day	14.1	14.1
	>30 g/day	2.3	2.3
	infrequent use	11.8	11.8
	missing	0.0	0.1
HRT use at baseline	no	81.6	81.6
	yes	18.4	18.4
	missing	0.0	0.3
Education	O-level college	69.8	69.7
	A-level college	7.0	7.0
	university	23.2	23.2
	missing	0.0	0.2
Socio-economic index	manual	38.3	38.1
	non-manual	54.1	54.2
	employer	7.6	7.6
	missing	0.0	0.0
Married or cohabiting	no	33.0	33.0
	yes	67.0	67.0
	missing	0.0	0.0
Bilateral oophorectomy	no	98.6	98.6
	yes	1.4	1.4
	missing	0.0	0.0
Age at menopause*	previous hysterectomy	0.5	0.5
	pre-/perimenopausal	34.0	34.0
	menopause ≤44	10.0	10.0
	menopause 45-54	49.6	49.6
	menopause ≥55	5.9	5.9
	missing	0.0	0.7
Parity	0	13.2	12.9
	1	21.4	21.5
	2	41.6	41.9
	3	17.0	17.1
	4 or more	6.7	6.7
	missing	0.0	1.6
Age at first childbirth	nullipara	13.3	12.9
	≤20	16.9	17.0
	21-25	35.6	35.9
	26-30	24.7	24.8
	≥31	9.6	9.4
	missing	0.0	1.7

Data is presented as valid column % and missing data is presented in total column % in categorical variables.
* Hysterectomized or pre-/perimenopausal women at baseline in separate categories. HRT = Hormone replacement therapy.

Supplementary Table S2. Distribution of established and potential risk factors for breast cancer in tertiles of allele score*.

		Low allele score	Intermediate allele score	High allele score
		Valid column %	Valid column %	Valid column %
Age at baseline**		57.5	57.3	57.4
Age at menarche**		13.6	13.6	13.6
Ever use of oral contraceptives	Never	50.8	50.6	51.0
	Ever	49.2	49.4	49.0
BMI	BMI <25	53.3	53.7	53.1
	BMI 25-30	33.3	32.8	33.0
	BMI ≥30	13.3	13.5	13.9
Alcohol intake	No alcohol	8.4	7.4	7.3
	<15 g/day	63.0	64.5	64.5
	15-30 g/day	13.8	14.4	14.0
	>30 g/day	2.4	2.3	2.4
	Infrequent use	12.4	11.4	11.8
HRT use at baseline	No	81.8	81.9	82.3
	Yes	18.2	18.1	17.7
Education	O-level college	70.0	69.8	69.6
	A-level college	7.1	6.9	6.9
	University	22.9	23.2	23.5
Socio-economic index	Manual	37.7	37.8	38.9
	Non-manual	54.5	55.1	53.2
	Employer	7.8	7.1	7.9
	Missing	0.9	1.1	1.0
Married or cohabiting	No	33.9	32.7	32.7
	Yes	66.1	67.3	67.3
Ooephorectomy, bilat	No	98.6	98.3	98.7
	Yes	1.4	1.7	1.3
Age at menopause***	Hysterectomy	0.5	0.8	0.4
	Pre-/peri	32.9	33.8	33.7
	≤44	9.7	9.9	10.1
	45-54	51.0	49.7	49.3
	≥55	5.9	5.8	6.5
Parity	0	13.6	12.8	12.5
	1	22.5	21.8	20.8
	2	40.9	41.7	42.6
	3	16.1	17.0	17.7
	4 or more	6.8	6.6	6.4
	Missing	1.6	1.5	1.8
Age at first childbirth	≤20	16.7	16.6	17.3
	21-25	34.7	36.0	36.5
	26-30	25.6	24.5	24.6
	≥31	9.4	10.0	9.1
	Nullipara	13.6	12.9	12.5
	Missing	1.7	1.7	1.9

Values are valid column % except for missing values that are total column %. Missing is not presented if <1% missing values in all columns. * The allele score is an externally weighted score based on GWAS-data of SNPs associated with increased serum and toenail selenium, see the articles method section for further details. ** Age is presented as mean years.
*** Hysterectomized or pre-/perimenopausal women at baseline in separate categories. HRT = Hormone replacement therapy.

Supplementary Table S3. Risk of breast cancer (BC) for women with low-, intermediate- and high allele score*. Total hazards are presented as BCs/100,000 person years (py). Low is reference for hazard ratios (HR) and 95 % confidence intervals (CI). Time at risk is person years from birth and endpoint is breast cancer diagnosis and other reasons for censoring is end of follow up, death, or emigration. Stratified for five SNPs. Values marked in bold indicate a significant interaction (p^i<0.05).

		Low allele score			Intermediate allele score			High allele score		
	Alleles	Women/ events	BCs/ 100,000py	HR	Women/ events	BCs/ 100,000py	HR (95% CI)	Women/ events	BCs/ 100,000py	HR (95% CI)
All		5491/661	154	1	5635/660	150	0.97 (0.87-1.08)	5303/635	154	1.00 (0.90-1.12)
GPX-1	C/C	2611/320	158	1	2758/334	155	0.98 (0.84-1.15)	2582/311	155	0.98 (0.84-1.15)
rs1050450	C/T	2391/288	154	1	2352/278	152	0.99 (0.84-1.17)	2245/277	158	1.03 (0.87-1.21)
	T/T	489/53	139	1	525/48	116	0.82 (0.56-1.21)	476/47	128	0.93 (0.63-1.38)
SOD-2	T/T	1343/160	153	1	1432/170	152	0.99 (0.80-1.22)	1338/168	162	1.06 (0.86-1.32)
rs4880	T/C	2805/344	157	1	2792/330	152	0.97 (0.83-1.13)	2619/306	150	0.95 (0.82-1.11)
	C/C	1343/157	150	1	1411/160	145	0.97 (0.77-1.20)	1346/161	154	1.03 (0.83-1.29)
SEPP1	G/G	3428/413	154	1	3473/408	150	0.97 (0.85-1.11)	3307/399	155	1.01 (0.88-1.16)
rs3877899	G/A	1827/219	153	1	1887/228	156	1.02 (0.85-1.23)	1780/208	149	0.97 (0.80-1.18)
	A/A	236/29	159	1	275/24	111	0.67 (0.39-1.16)	216/28	171	1.10 (0.66-1.85)
SEPP1	G/G	2503/279	143	1	2544/280	141	0.99 (0.84-1.17)	2323/305	169	1.20 (1.02-1.41)
rs7579	G/A	2419/317	168	1	2498/309	158	0.94 (0.80-1.10)	2411/272	144	**0.85 (0.73-1.00)**
	A/A	569/65	146	1	593/71	154	1.06 (0.76-1.48)	569/58	132	0.90 (0.63-1.29)
GPX-4	G/G	1842/217	151	1	1789/226	162	1.08 (0.90-1.30)	1774/208	151	1.01 (0.83-1.22)
rs713041	G/A	2649/325	158	1	2846/323	146	0.92 (0.79-1.07)	2594/311	154	0.97 (0.83-1.14)
	A/A	1000/119	152	1	1000/111	142	0.94 (0.72-1.21)	935/116	160	1.06 (0.82-1.37)

*The allele score is an externally weighted score based on GWAS-data of SNPs associated with increased serum and toenail selenium, see the method section in the article for further details.

Supplementary Table S4. Risk of breast cancer (BC) for women with low-, intermediate- and high dietary intake of selenium. Hazard presented as BCs/100,000 person years (py). Low is reference for Hazard Ratios (HR) and 95% confidence intervals (CI). Time at risk is years from start of cohort and endpoint is first time BC diagnosis. Other reasons for censoring is end of follow up, death, or emigration. Stratified for five SNPs. Values marked in bold indicates a significant interaction ($p^{i}<0.05$).

		Low selenium intake			Intermediate selenium intake				High selenium intake			
	Alleles	Women/ events	BCs/ 100,000py	HR	Women/ events	BCs/ 100,000py	HR[1] (95% CI)	HR[2] (95% CI)	Women/ events	BCs/ 100,000py	HR[1] (95% CI)	HR[2] (95% CI)
ALL		5296/499	435	1	5299/430	377	0.88 (0.77-1.00)	0.86 (0.76-0.98)	5296/489	432	1.02 (0.90-1.15)	0.97 (0.85-1.10)
GPX-1	C/C	2597/236	419	1	2527/224	411	0.99 (0.83-1.20)	0.98 (0.81-1.17)	2568/246	449	1.09 (0.91-1.30)	1.06 (0.88-1.27)
rs1050450	C/T	2214/216	450	1	2308/176	354	0.80 (0.66-0.98)	0.78 (0.64-0.95)	2228/213	447	1.02 (0.85-1.24)	0.95 (0.79-1.16)
	T/T	485/47	451	1	464/30	302	0.68 (0.43-1.08)	-	500/30	278	**0.63 (0.40-1.00)**	**-**
SOD-2	T/T	1319/ 118	414	1	1296/109	395	0.99 (0.76-1.28)	0.98 (0.75-1.27)	1339/112	388	0.96 (0.76-1.25)	0.92 (0.70-1.19)
rs4880	T/C	2684/ 264	455	1	2679/212	365	0.81 (0.68-0.97)	0.80 (0.67-0.96)	2595/246	442	0.99 (0.83-1.18)	0.93 (0.78-1.11)
	C/C	1293/ 117	413	1	1324/109	383	0.94 (0.73-1.22)	0.90 (0.69-1.18)	1362/131	456	1.12 (0.87-1.44)	1.07 (0.83-1.37)
SEPP1	G/G	3279/ 308	433	1	3283/260	369	0.86 (0.73-1.02)	0.85 (0.72-1.00)	3306/312	440	1.04 (0.89-1.22)	0.98 (0.83-1.15)
rs3877899	G/A	1767/171	447	1	1802/154	393	0.89 (0.72-1.11)	0.88 (0.70-1.09)	1750/155	416	0.95 (0.76-1.18)	0.91 (0.73-1.14)
	A/A	250/20	364	1	214/16	362	1.02 (0.53-1.98)	-	240/22	436	1.23 (0.67-2.26)	-
SEPP1	G/G	2382/220	426	1	2347/177	352	0.85 (0.70-1.04)	0.83 (0.68-1.02)	2384/210	413	1.01 (0.83-1.22)	0.96 (0.79-1.16)
rs7579	G/A	2373/231	448	1	2364/210	411	0.92 (0.77-1.11)	0.91 (0.75-1.10)	2366/232	457	1.03 (0.86-1.23)	0.97 (0.81-1.17)
	A/A	541/48	416	1	588/43	338	0.82 (0.55-1.24)	-	546/47	406	1.00 (0.67-1.49)	-
GPX-4	G/G	1737/167	442	1	1751/146	388	0.90 (0.72-1.12)	0.88 (0.71-1.11)	1725/146	395	0.91 (0.73-1.14)	0.86 (0.69-1.08)
rs713041	G/A	2651/241	419	1	2589/206	369	0.89 (0.74-1.08)	0.87 (0.72-1.05)	2596/259	466	1.14 (0.95-1.36)	1.10 (0.92-1.31)
	A/A	908/91	466	1	959/78	378	0.82 (0.61-1.11)	0.81 (0.59-1.10)	975/84	405	0.89 (0.66-1.20)	0.85 (0.63-1.15)

Mean total selenium intake for tertiles; Low: 26 µg/day, Intermediate: 35 µg/day, High: 69 µg/day.

1: Adjusted for age at baseline

2: Adjusted for age at baseline, education, socioeconomic index, marital status, age at menarche, age at menopause, number of children, age at childbirth, use of oral contraceptives, oophorectomy, BMI, hormone replacement therapy, alcohol intake.

No HR is presented if the analysis was unstable for the number of events compared to number of adjustment categories.

Supplementary Table S5. Risk of breast cancer for women with low-, intermediate- and high serum selenium with low as reference for odds ratios (ORs) and 95% confidence intervals (CI). Time at risk is person years from start of cohort and endpoint is breast cancer diagnosis and other reasons for censoring is end of follow up, death, or emigration. Stratified for five SNPs. No significant interaction (p^i<0.05) was seen from the SNPs.

		Serum selenium low		Serum selenium intermediate			Serum selenium high		
	Alleles	Women/events	OR	Women/events	OR[1] (95% CI)	OR[2] (95% CI)	Women/events	OR[1] (95% CI)	OR[2] (95% CI)
ALL		677/348	1	685/367	1.11 (0.90-1.37)	1.05 (0.84-1.31)	675/332	0.94 (0.76-1.16)	0.85 (0.68-1.07)
GPX 1	C/C	358/182	1	306/165	1.15 (0.85-1.56)	1.08 (0.78-1.49)	331/162	0.94 (0.70-1.27)	0.85 (0.62-1.17)
rs1050450	C/T	264/142	1	317/173	1.05 (0.76-1.46)	0.92 (0.65-1.30)	286/142	0.87 (0.62-1.22)	0.76 (0.93-1.09)
	T/T	55/24	1	62/29	1.15 (0.55-2.38)	-	58/28	1.23 (0.59-2.60)	-
SOD 2	T/T	180/92	1	145/76	1.11 (0.71-1.72)	0.80 (0.49-1.32)	161/75	0.86 (0.56-1.33)	0.69 (0.43-1.11)
rs4880	T/C	324/170	1	355/195	1.11 (0.82-1.50)	1.06 (0.78-1.46)	349/177	0.94 (0.69-1.27)	0.83 (0.60-1.15)
	C/C	173/86	1	185/96	1.14 (0.75-1.73)	1.19 (0.76-1.86)	165/80	1.00 (0.65-1.54)	1.05 (0.66-1.67)
SEPP1	G/G	405/204	1	409/217	1.14 (0.86-1.50)	1.02 (0.72-1.46)	441/211	0.93 (0.71-1.22)	0.93 (0.64-1.35)
rs3877899	G/A	237/126	1	249/133	1.02 (0.72-1.46)	0.94 (0.64-1.36)	216/110	0.93 (0.64-1.35)	0.84 (0.56-1.25)
	A/A	35/18	1	27/17	1.60 (0.58-4.48)	-	18/11	1.51 (0.47-4.84)	-
SEPP1	G/G	301/151	1	298/167	1.33 (0.96-1.85)	1.19 (0.84-1.68)	306/139	0.87 (0.63-1.20)	0.77 (0.54-1.08)
rs7579	G/A	309/162	1	313/163	0.99 (0.72-1.35)	0.98 (0.70-1.37)	298/158	1.02 (0.74-1.41)	0.97 (0.69-1.37)
	A/A	67/35	1	74/37	0.96 (0.49-1.88)	-	71/35	0.94 (0.48-1.85)	-
GPX 4	G/G	232/122	1	216/104	0.85 (0.58-1.23)	0.81 (0.54-1.20)	221/110	0.91 (0.63-1.32)	0.85 (0.57-1.26)
rs713041	G/A	331/168	1	342/195	1.31 (0.97-1.78)	1.22 (0.89-1.69)	336/162	0.93 (0.68-1.26)	0.80 (0.58-1.12)
	A/A	114/58	1	127/68	1.12 (0.67-1.86)	1.02 (0.58-1.78)	118/60	1.00 (0.60-1.68)	0.82 (0.46-1.45)

Mean tertile serum selenium levels, Low: 75ng/ml, Intermediate: 90ng/ml, High: 108ng/ml.

1: Adjusted for age at baseline.

2: Adjusted for age at baseline, education, socioeconomic index, marital status, age at menarche, age at menopause, number of children, age at childbirth, use of oral contraceptives, oophorectomy, BMI, hormone replacement therapy, alcohol intake.

No OR is presented if the analysis was unstable for the number of events compared to number of adjustment categories.

Supplementary Figure S1. Frequency tables for number of alleles reported in continouos values (imputed probability).

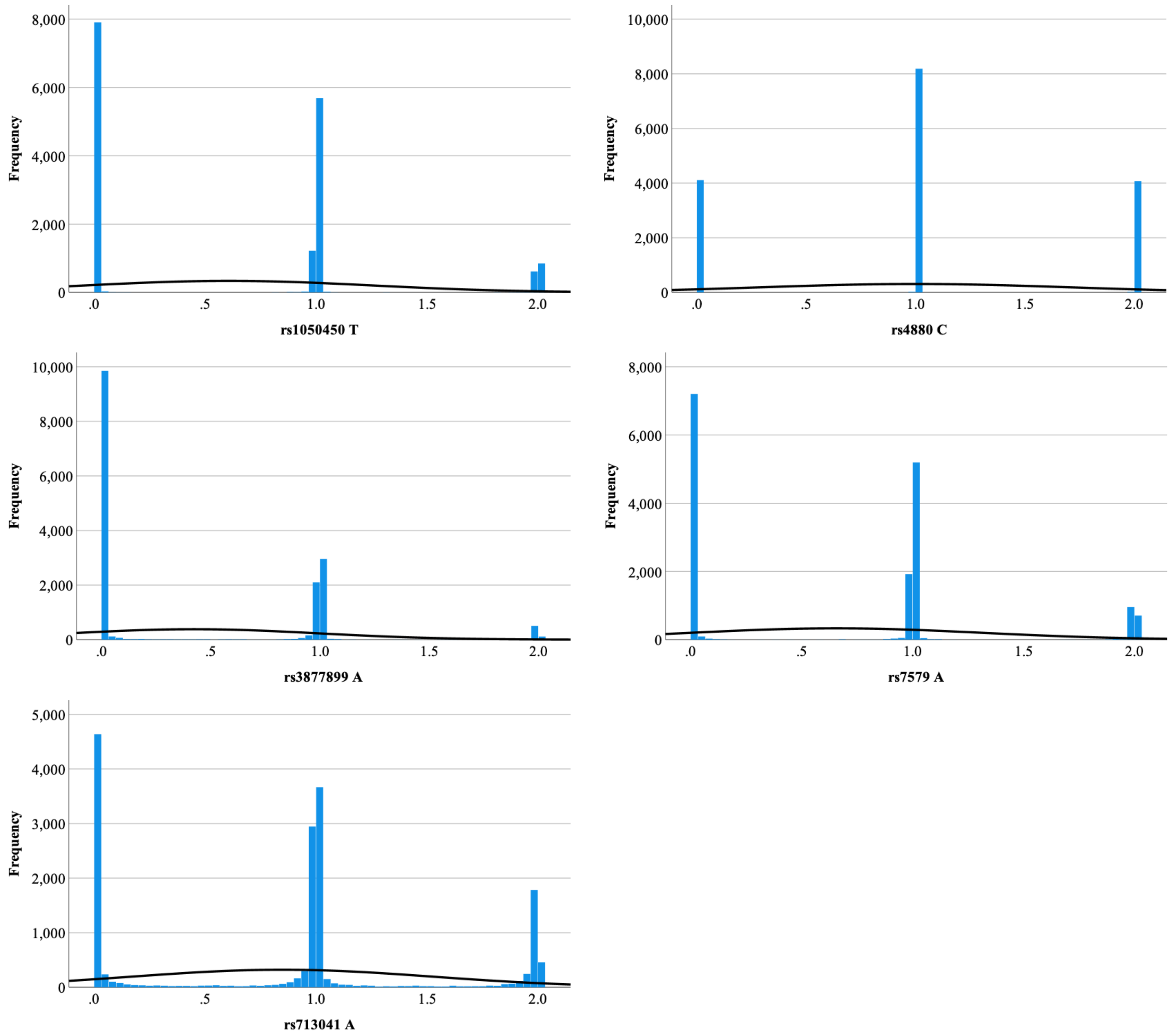

Supplementary Figure S2. Kaplan-Meier curves for tertiles of Se exposure and breast cancer risk.

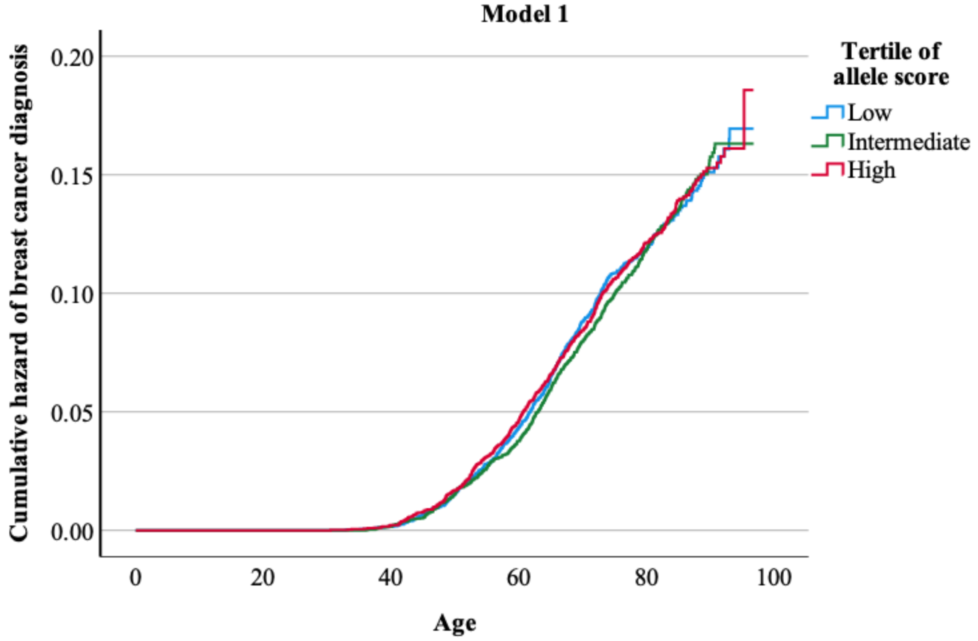

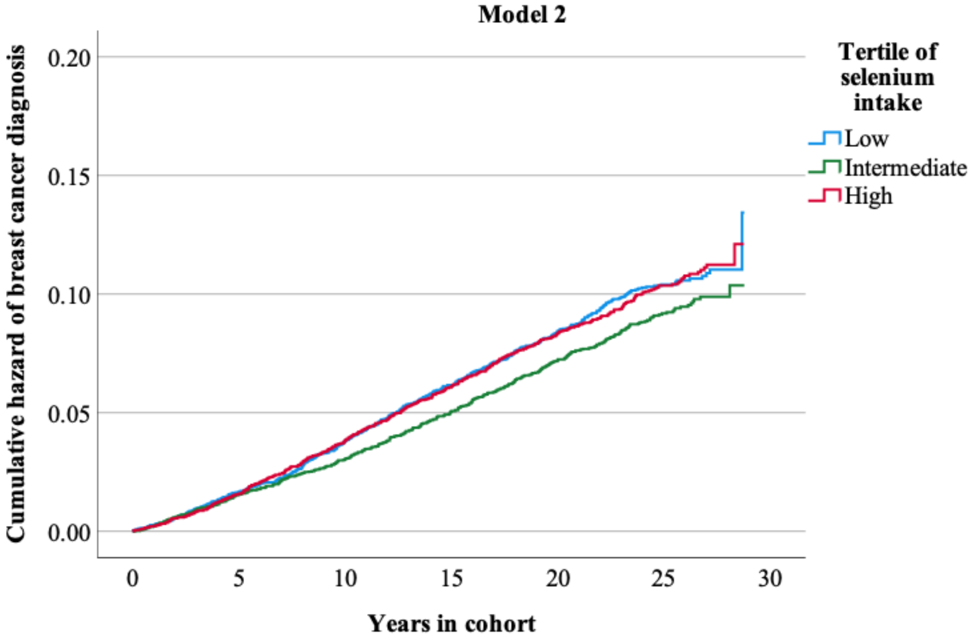

Supplement: Supplementary file 1 [file nutrients-14-00826-s001.zip › nutrients-1562237-supplementary.pdf]
